# Correlative Evaluation of Mental and Physical Workload of Laparoscopic Surgeons Based on Surface Electromyography and Eye-tracking Signals

**DOI:** 10.1038/s41598-017-11584-4

**Published:** 2017-09-11

**Authors:** Jian-Yang Zhang, Sheng-Lin Liu, Qing-Min Feng, Jia-Qi Gao, Qiang Zhang

**Affiliations:** 1 Department of Medical Engineering, Union Hospital, Tongji Medical College, Huazhong University of Science and Technology, Wuhan, 430022 China; 2 School of Computer and Information Technology, Nanyang Normal University, Nanyang, Henan P.R. China; 3 Healthcare Ergonomics Lab, Union Hospital, Tongji Medical College, Huazhong University of Science and Technology, Wuhan, 430022 China

## Abstract

Surgeons’ mental and physical workloads are major focuses of operating room (OR) ergonomics, and studies on this topic have generally focused on either mental workload or physical workload, ignoring the interaction between them. Previous studies have shown that physically demanding work may affect mental performance and may be accompanied by impaired mental processing and decreased performance. In this study, 14 participants were recruited to perform laparoscopic cholecystectomy (LC) procedures in a virtual simulator. Surface electromyography (sEMG) signals of the bilateral trapezius, bicipital, brachioradialis and flexor carpi ulnaris (FCU) muscles and eye-tracking signals were acquired during the experiment. The results showed that the least square means of muscle activity during the LC phases of surgery in an all-participants mixed effects model were 0.79, 0.81, and 0.98, respectively. The observed muscle activities in the different phases exhibited some similarity, while marked differences were found between the forearm bilateral muscles. Regarding mental workload, significant differences were observed in pupil dilation between the three phases of laparoscopic surgery. The mental and physical workloads of laparoscopic surgeons do not appear to be generally correlated, although a few significant negative correlations were found. This result further indicates that mental fatigue does markedly interfere with surgeons’ operating movements.

## Introduction

Surgeons encounter musculoskeletal strain and disorders resulting from long periods of muscle tension and awkward poses^1–3^. Injuries to surgeons include pain in specific areas of the body, vertebral disk prolapse and carpal tunnel syndrome^4,5^. These issues are closely related to the mental and physical workloads of surgeons during surgery^2^. In terms of mental workload, surgeons can suffer from impaired concentration and slow reactions after long operations. Furthermore, job dissatisfaction of surgeons is considered to be significantly associated with burnout^6^. As muscle fatigue and attention deficit may contribute to failed surgeries, risk monitoring and risk reduction measures should be implemented if a surgeon is experiencing physical or mental overload or fatigue. Surgeons’ mental and physical workloads have been a focus of operating room (OR) ergonomics over the last few decades.

To assess surgeons’ mental and physical workloads, laparoscopic box trainers and virtual reality simulators are usually employed and are comparable in most aspects^7,8^. Some studies have suggested that virtual simulators may be more reliable and convenient, and peg transfer, ball pick-and-drop, and cutting and suturing are commonly simulated procedures^2^. Most previous studies have focused on either mental workload or physical workload but have seldom performed comparative analyses^9–12^. Taking into account psychophysiological causes and related literature, the relationship between the two types of workloads should be considered.

Metrics used to assess surgeon workload include subjective measures of workload, physiological indices of workload, objective performance, and other methods including comprehensive evaluations. Scales and questionnaires, such as the NASA Task Load Index scale^13^ and the Subjective Workload Assessment Technique scale, have become among the most popular tools, especially for surgical procedures^14–16^. Various physiological indices, such as heart rate, blood pressure, eye movements, EMG, and EEG signals, etc. refs^17,18^, change corresponding to changes in workload; heart rate is generally used to evaluate body load, and eye movements, and EEG are generally used to assess mental workload. In particular, EEG can characterize the dynamics of functional coupling among different brain areas across surgeons performing laparoscopic tasks with different approaches^19^. In addition, workload status can be deduced through tasks and the associated performance. These different workload evaluation methods each have their own advantages, and physiological indices of workload are more prominent in accuracy and objectivity.

A previous study presented an interesting finding that the mental workload of bank staff is significantly correlated with musculoskeletal disorders^20^. The mental workload of nurses is also associated with musculoskeletal disorders^21^. This previously reported conclusion is based on different types of work and different work contents, and those surveyed enjoyed certain autonomy while working^22,23^. In contrast, considering a surgeon’s workload, the equipment used, the working time and the processes are severely restricted during an operation. In addition, surgeons must meet high mental and physical demands, have high operation accuracy, and make accurate judgements and decisions. Mental status is associated with muscle activity in some work situations. Schleifer *et al*.^24^ discovered that mental stress results in increased EMG activity of the upper limbs during computer work. With the differential changes in heart period and end-tidal carbon dioxide in differential working conditions, mental stress elicits more psychophysiological activation, and less effects are attributed to the biomechanical demands of work. Furthermore, high mental workload tasks predispose individuals to increased psychological and physiological activation. Mental fatigue also influences muscle endurance, recovery and EMG activity^25,26^.

The interactive effects of mental and physical workload have received growing attention, and negative correlations between mental workload and physical workload have been reported^22^. In the foregoing cited study, subjective self-report rating assessment tools, the Borg CR10 Scale and NASA-TLX, were adopted to assess physical and mental workloads, respectively. The dual-task methodology consisted of a physical lifting task (no load, 8%,14% and 20% of body mass) and a mental arithmetic task (no load, addition, subtraction, and multiplication) with a total of 15 combinations of conditions. This approach has also been commonly used in other studies^23,24,27^. Compared with the interactive effects of mental and physical workloads that have been assessed for different types of tasks, laparoscopic surgeries contain both heavier mental and physical loads.

## Results

The calculated muscle activity levels are shown in Tables 1, 2 and 3. Descriptive statistics are shown in Table 1, the fixed effects of the characteristics on the results are given in Table 2, and statistics for the various phases are listed in Table 3. The physical workload patterns during the 3 phases were generally similar, with minor differences between the left and right trapezius muscle and bicipital muscle and large differences in the brachioradialis and FCU. The most significant finding was that the activities of the eight muscles in the AC phase (disinterring the bile duct and the cystic artery) and SC phase (sealing and cutting the bile duct and the cystic artery) were quite similar (mean difference = 0.02, p < 0.05) and significantly lower than the muscle activities in the DI phase (detaching the gallbladder from the hepatic bed and inspecting the hepatic bed) (p = 0.01 and 0.03, respectively). Interestingly, the left brachioradialis %MVC was nearly twice that of the right brachioradialis, and the bilateral FCU exhibited the opposite trend, with the exception of during the DI phase.

**Table 1:** Description of four upper extremity muscles. The %MVC values for all combinations are presented as the mean (standard deviation).

Phase	Trapezius		Bicipital		Brachioradialis		FCU	
	Left	Right	Left	Right	Left	Right	Left	Right
AC	0.88 (0.49)	1.01 (0.8)	0.84 (0.56)	0.68 (0.26)	1.01 (1.4)	0.45 (0.25)	0.55 (0.55)	0.89 (0.86)
SC	0.83 (0.36)	0.96 (0.78)	0.94 (0.66)	0.77 (0.31)	1.01 (1.3)	0.45 (0.23)	0.53 (0.29)	0.99 (0.78)
DI	1.12 (0.64)	1.07 (0.78)	1.1 (0.65)	0.94 (0.59)	1.18 (1.26)	0.53 (0.38)	0.99 (0.44)	0.91 (0.6)

**Table 2:** Fixed effects of characteristics based on results of a mixed model.

Characteristics	Class	Coefficient	S.E.	D.F.	t-value	P
**Muscle**	Trapezius	−0.08	0.19	80.2	−0.43	0.67
	Bicipital	−0.53	0.19	80.2	−2.75	0.01
	Brachioradialis	−0.22	0.19	80.2	−1.12	0.27
	FCU	ref	—	—	—	—
**Location**	Left	−0.07	0.19	80.2	−0.35	0.73
	Right	ref				
**Phase**	AC	−0.19	0.07	265	−2.88	0.004
	SC	−0.17	0.07	265	−2.56	0.01
	DI	ref				
**Muscle** * **location**	Trapezius * Left	−0.17	0.15	265	−1.13	0.26
	Bicipital * Left	0.65	0.15	265	4.3	<0.001
	Brachioradialis * Left	0.23	0.15	265	1.53	0.13

**Table 3:** Least square means and multiple comparisons of the LC phases in an all-participants mixed effects model.

Phase	Least square mean	S.E.	P-value	Multiple comparison					
				Mean difference			Bonferroni adjustment for P-value		
				AC	SC	DI	AC	SC	DI
AC	0.79	0.08	<0.001	—	−0.02	−0.19	—	1.00	0.01
SC	0.81	0.08	<0.001	—	—	−0.17	—	—	0.03
DI	0.98	0.08	<0.001	—	—	—	—	—	—

**Figure 1:**
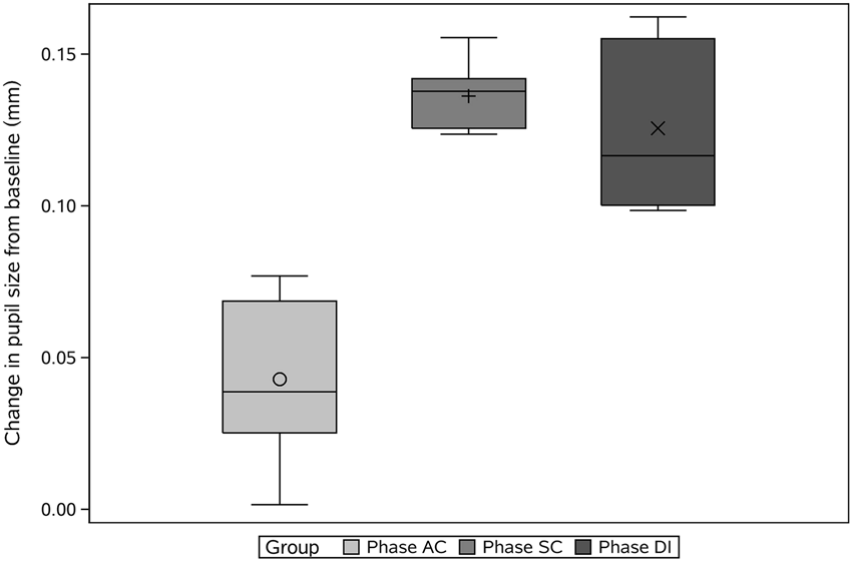
Change in pupil size from baseline during the 3 phases of laparoscopic cholecystectomy surgery.

Figure 1 demonstrates the change in the participants’ pupil diameter during the 3 LC phases. The extent of pupil dilation during the SC phase (mean = 0.12, median = 0.13) and DI phase (mean = 0.13, median = 0.13) was less than that in the DI phase (mean = 0.05, median = 0.04). Moreover, the pupil diameter increased during each individual phase.

The results of a correlation analysis between sEMG measurements and eye-tracking is shown in Table 4. We found that the sEMG and eye-tracking measurements during the different phases were uncorrelated. The activities of the left brachioradialis and the left FCU in the SC phase were significantly negatively correlated with mental workload (r = −0.68, p = 0.01 and r = −0.53, p = 0.05).

**Table 4:** Correlation analysis between mental and physical workload during the 3 LC phases.

Muscle	location	AC Phase		SC Phase		DI Phase	
		Coefficient	P-value	Coefficient	P-value	Coefficient	P-value
Trapezius	left	0.05	0.85	−0.05	0.85	−0.15	0.62
	right	0.07	0.81	0.19	0.52	0.09	0.76
Bicipital	left	−0.35	0.23	−0.27	0.34	−0.32	0.26
	right	0.14	0.63	−0.26	0.38	−0.14	0.64
Brachioradialis	left	−0.33	0.25	−0.68	0.01	−0.37	0.19
	right	−0.27	0.34	−0.5	0.07	−0.43	0.13
FCU	left	−0.32	0.26	−0.53	0.05	−0.35	0.22
	right	0.14	0.63	0.02	0.95	−0.1	0.73

## Discussion

Our study is the first to address the significant concern regarding the relationship between the two types of workloads on laparoscopic surgeons. The experimental platform and tasks were carefully considered. An LC surgery was divided into 3 approximately equal phases in terms of the time and process. This partitioning method was effective for our study and is convenient for acquiring and comparing sEMG and eye-tracking signals^18^.

For evaluating physical workload, similarities in muscle activity among different phases can be determined. This phenomenon can be explained by similar gestures and movements. Notably, we found a difference between the sides of the brachioradialis and the FCU. Fine operations are usually carried out by the dominant hand (i.e., the right hand), and fine movements rely heavily on the wrist and fingers. The left brachioradialis was employed more during usual motions, while the FCU was utilized for finer motions, which reflects muscle movement compensation.

The mean changes in pupil size during the 3 phases are shown in Fig. 1. In this experiment, the pupil was able to characterize mental workload according to expectations. A high mental workload within a short time does not cause mental fatigue and thus does not result in a cumulative effect, which is consistent with the conclusions of other studies^28^. Other factors influencing pupil size include anxiety, stress, fatigue, and intelligence. In our experimental design, we attempted to eliminate the effects of these factors on pupil size through various methods: allowing the participants to relax, preventing participants from performing tests in a fatigued state, and adjusting lighting brightness of the scene.

Our experimental findings suggest that the mental and physical workloads of the laparoscopic surgeon were non-synchronous and were generally negatively correlated, although insignificant. Mental workload during low-level static work has been verified to adversely affect muscle activity. Laparoscopic surgery involves low-level strength and high-level mental workload. The surgeons’ physical workloads in the AC and SC phases were almost equal and were much lower than the physical workload during the DI phase. In contrast, the surgeons’ mental workload in the AC phase was lower than the mental workload in the SC and DI phase, which corresponded to similar workload levels. The relationship between the workloads can be explained using physiology. Studies of the brain have indicated that mental fatigue and physical fatigue are closely linked. When people are physically fatigued, blood oxygenation in the bilateral prefrontal cortex is reduced, which aggravates mental fatigue^29^. Muscle activity is directly related to neural activity, as proven by neuroimaging techniques, and the brain possesses a self-adjusting function to maintain physical performance, even when falling into a state of fatigue^30–32^.

Our experimental results showed that there was no significant negative correlation between the workloads, which is not entirely consistent with previous studies. We attributed this discrepancy to the following reasons: (1) surgical procedures involve both mental and physical workloads, unlike the individual mental and physical tasks employed in previous studies, and the two workloads are not completely independent. Co-existence of the workloads indicates that their relationship is not entirely interactive. (2) We selected a representative group of muscles as research targets but did not include all muscles used during an operation performed by a surgeon, which may result in bias.

Objective evaluations of the workload and ergonomics of laparoscopic surgeons are vital and meaningful. More studies are needed to compensate for the limitations of this study. Physical and mental workload levels are complex and cannot be characterized in a general manner. Multi-means, indices and subjective methods combined with objective techniques will be the most promising approaches going forward^33^. Workload threshold and ergonomics guidelines should be elaborated to prevent ergonomic problems during laparoscopic surgery.

This experiment was based on a simulation, which holds obvious limitations compared to actual operations. Participants may not be as careful when using a simulator because they may perceive that there will be another chance to repeat the procedure without repercussions. Another limitation of this study was that the LC operation duration was not long enough to induce surgeon fatigue, and therefore, surgeon workloads during a fatigued state could not be evaluated. We plan to study the working status of laparoscopic surgeons and its impact on operation safety and outcomes under different workload conditions in the near future. The concept of a surgeon’s total workload should be established, which would provide a general description of the physician’s fatigue status for quantitative and intuitive monitoring.

## Methods

### Participants

The procedures of this study were carried out in accordance with approved guidelines. This study was approved by the Ethics Committee of Tongji Medical College, Huazhong University of Science and Technology (IORG No: IORG0003571), and was performed in a simulated operating room with proper lighting conditions and other requirements for an operating environment, according to related standards and manufacturer recommendations. Informed consent was obtained from all participants. In this study, 14 male volunteers were recruited. Four of the participants were laparoscopic surgeons, and the other 10 were predoctoral students. All individuals had laparoscopic surgery experience or training experience and were familiar with the experimental platform prior to the experiment. All participants were right-handed, and they ranged in age from 25 to 35 years (mean age = 28.7, SD = 3.8). The participants’ body mass index (BMI) and elbow height were measured and used as references to adjust the experimental set-up.

### Experimental platform and tasks

The experiment was executed in a laparoscopic virtual simulator, which can provide feedback on the operation performance of the participants, including haptic feedback. Statistics of the participants performance on the task were provided when the task was completed.

LC is one of the most common laparoscopic surgeries. LC has been used in many studies as a sample procedure to study the working status of laparoscopic surgeons with respect to OR ergonomics^34–36^. In previous research, surgical videos, combined with other tools such as rapid upper limb assessment (RULA), have been employed to study physician gestures and stress statics^36,37^. In contrast to other studies, our study aimed to explore both the physical and mental workloads of laparoscopic surgeons in different LC phases and the correlative relationship between these workloads. All participants were required to complete an LC surgery using the simulator. According to the operation process and the operation simulator’s setting, the LC should be completed via the following five phases: Phase 1: create the pneumoperitoneum and place the trocars; phase 2: based on the anatomy of Calot’s triangle, disinter the bile duct and the cystic artery (AC phase); phase 3: seal and cut the bile duct and the cystic artery (SC phase); phase 4: detach the gallbladder from the hepatic bed and inspect the hepatic bed (DI phase); and phase 5: remove the gallbladder and complete the operation. Phase 1 and phase 5 were executed automatically, and the participants were required to complete the AC, SC and DI phases. We advised the participants to allocate 5 minutes to each of the three phases and to finish the surgery in 15 minutes, if possible. Intervals of approximately 3 minutes were included between the phases to allow the participants’ muscles to relax and to provide feedback.

### Workload assessment protocol

#### Data analysis

An overall 14 × 4 × 2 × 3 (14 participants × 4 muscles × 2 hand sides × 3 phases) analysis of variance was used to analyse the data. A mixed effect model was used for statistical analysis in SAS 9.4, with the significance level set at p = 0.05. Variables with random effects were selected based on the smallest Akaike information criterion (AIC) and the Bayesian information criterion (BIC), with a positive definite G matrix for the intercept, muscles, location and phase.

#### Eye-tracking data

According to many studies, mental workload can be evaluated by participants’ eye movements, particularly pupil dilatation^38–40^. The Tobii Glasses 2 Eye Tracker (Tobii Technology, Danderyd, Sweden) was used as an eye-tracking instrument in our study. Before starting the procedure, the participants were equipped with the eye tracker and asked to stare at black dots printed on a paper card for the calibration process. The physiological parameters of the participants’ eyes were recorded during the calibration process.

Laparoscopic surgery requires a high degree of attention, and therefore, eye movement is more able to reflect the physiological state of the surgeon and surgical conditions. Here, pupil size was analysed as the focal index to measure mental workload during operation. In the 1960s, pupil dilation was found to be sensitive to task difficulty and workload^41,42^. Pupil dilation can be used as a peripheral indicator of brain noradrenergic activity and mental workload in a testing situation. The measurement of pupil diameter has been deemed a promising method for assessing mental workload^38,43,44^. Task-evoked pupillary responses (TEPRs) have been suggested for exploring the inherent relationship between a task and pupillary dilation^44^. Generally, larger pupil sizes indicate greater mental workload^45–47^. In this study, the baseline pupil size (initial diameter) was assessed after the calibration period^48^. A change in pupil size from baseline, measured as the mean pupil diameter change (MPDC), was observed, consistent with the expected effect.

#### sEMG data

Physical workload was evaluated by surface myoelectricity, which was captured using a Delsys Trigno Lab sEMG system (Delsys, Inc., Boston, MA) and analysed with standard software. The muscle groups analysed included the bilateral trapezius, biceps, brachioradialis, and FCU. The sEMG sampling frequency was 512 Hz. These data were full-wave rectified and then filtered to obtain a spectrum band ranging from 20 Hz to 250 Hz.

At the beginning of the experimental session, we measured the maximum voluntary contraction (MVC) of each target muscle and normalized the sEMG data to the MVC during data processing^49^. In this study, we used %MVC, the percentage of MVC, as a measure of muscle workload and characterized the level of muscle contraction per unit time^2^. For data processing, iEMG was obtained by first integrating the sEMG; the ratio of iEMG to MVC was taken as %MVC^2^ ^49^.

1 $$ \% {\rm{MVC}}=\frac{{\rm{iEMG}}}{{\rm{MVC}}}\times 100 \% $$

## Conclusions

Observations of surgeons operating during different phases of LC and measurements of their mental and physical workload indicated that the two workloads are non-synchronous, with a general non-significant negative correlation. This study evaluated the workload imposed on surgeons during laparoscopic surgery by physiological (sEMG and eye movement) analysis and objectively demonstrated that while some laparoscopic phases require equal levels of physical work and others do not, significant disparities exist among the mental workloads of those phases. Synthetic and dynamic monitoring of surgeon workload levels is thus highly important in OR ergonomics.
