# Author Correction: Correlative Evaluation of Mental and Physical Workload of Laparoscopic Surgeons Based on Surface Electromyography and Eye-tracking Signals

**DOI:** 10.1038/s41598-018-23759-8

**Published:** 2018-04-25

**Authors:** Jian-Yang Zhang, Sheng-Lin Liu, Qing-Min Feng, Jia-Qi Gao, Qiang Zhang

**Affiliations:** 1 Department of Medical Engineering, Union Hospital, Tongji Medical College, Huazhong University of Science and Technology, Wuhan, 430022 China; 2 School of Computer and Information Technology, Nanyang Normal University, Nanyang, Henan P.R. China; 3 Healthcare Ergonomics Lab, Union Hospital, Tongji Medical College, Huazhong University of Science and Technology, Wuhan, 430022 China

Correction to: *Scientific Reports* 10.1038/s41598-017-11584-4, published online 11 September 2017

In the original version of this Article, Affiliations 1 and 3 were incomplete. The correct affiliations are listed below.

Affiliation 1:

Department of Medical Engineering, Union Hospital, Tongji Medical College, Huazhong University of Science and Technology, Wuhan 430022, China.

Affiliation 3:

Healthcare Ergonomics Lab, Union Hospital, Tongji Medical College, Huazhong University of Science and Technology, Wuhan 430022, China.

These errors have now been corrected in the PDF and HTML versions of the Article.

